# Retroviral and Lentiviral Vectors for the Induction of Immunological Tolerance

**DOI:** 10.6064/2012/694137

**Published:** 2012-07-18

**Authors:** Inès Dufait, Therese Liechtenstein, Alessio Lanna, Christopher Bricogne, Roberta Laranga, Antonella Padella, Karine Breckpot, David Escors

**Affiliations:** ^1^Division of Infection and Immunity, Rayne Institute, University College London, 5 University Street, London, WC1E 6JF, UK; ^2^Department of Physiology and Immunology, Medical School, Free University of Brussels, Laarbeeklaan 103, 1090 Jette, Belgium

## Abstract

Retroviral and lentiviral vectors have proven to be particularly efficient systems to deliver genes of interest into target cells, either in vivo or in cell cultures. They have been used for some time for gene therapy and the development of gene vaccines. Recently retroviral and lentiviral vectors have been used to generate tolerogenic dendritic cells, key professional antigen presenting cells that regulate immune responses. Thus, three main approaches have been undertaken to induce immunological tolerance; delivery of potent immunosuppressive cytokines and other molecules, modification of intracellular signalling pathways in dendritic cells, and de-targeting transgene expression from dendritic cells using microRNA technology. In this review we briefly describe retroviral and lentiviral vector biology, and their application to induce immunological tolerance.

## 1. Introduction

Viruses are obligate intracellular parasites which have evolved to transfer their genetic material to infected cells and use their biosynthetic machinery to replicate, encapsidate, and package their genome. Virus particles are usually secreted so they can infect neighbouring cells and start a new infectious cycle. The discovery that viruses could also incorporate and transmit genes of cellular origin opened the possibility of using them as tools to genetically modify cells. The engineering of virus vectors has allowed the development of gene vaccines for a wide range of infectious diseases and cancer. It is relatively easy to raise effective immune responses against transgenes encoded in virus vectors. After all, the innate immune system contains all necessary mechanisms to recognise virus particles leading to its strong and speedy activation. As such, virus particles act as “natural” adjuvants. However, their capacity to activate the immune system restricts their applicability for the treatment of autoimmune disorders. In addition, their application for the correction of a genetic disease is also limited because of their immunogenicity. Transgene-specific immune responses severely limit the success of gene therapy. However, despite all disadvantages, virus vectors have been used to establish strong antigen-specific immune suppression. This has been achieved by the expression of immunosuppressive cytokines, modulators of intracellular signalling pathways, and the incorporation of microRNA targets.

## 2. Retrovirus and Lentivirus Vectors

There is an ever-growing list of different virus species that are being used as virus vectors. Amongst those, the most extensively used are adenoviruses, adeno-associated viruses, and poxviruses. Interestingly, those belonging to the Retroviridae family are possibly within the most successful. Retrovirus vectors such as those based on Moloney mouse leukemia virus (MLV) were amongst the first to be engineered [1]. They have also been the first to be successfully applied in human gene therapy for the correction of genetic disorders [2–5]. In recent years, lentivectors have strongly appeared in biomedicine as an alternative to *γ*-retrovirus vectors. As their retrovirus cousins, lentivectors are devoid of viral proteins, stably incorporate their genome into the host cell, and lead to long-term transgene expression. In addition, unlike the simple retroviruses, they can transduce nondividing cells [6]. This characteristic opens up their application in gene therapy to genetically target highly differentiated cells such as neurons and dendritic cells.

**Figure 1:**
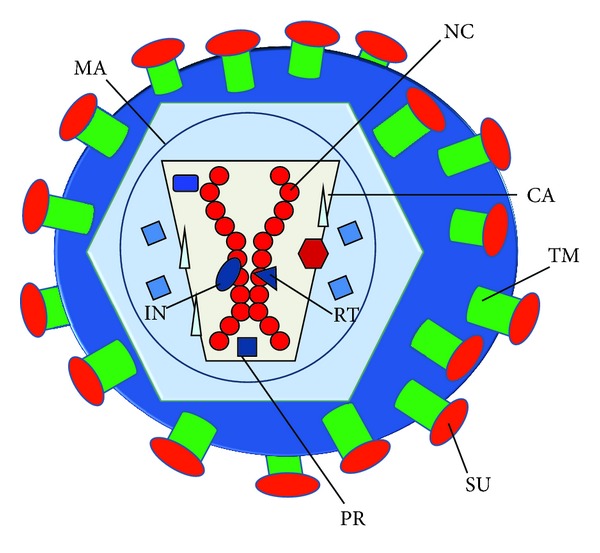
General structure of the retrovirion. The structure of the retrovirion is schematically shown in the figure. The retrovirus contains a diploid RNA genome associated to the nucleocapsid protein (NC), forming the nucleocapsid. This nucleocapsid is associated to structural proteins involved in the retrotranscription (RT), integration (integrase, IN), and virion maturation (protease, PR). All these are enclosed by the capsid (CA) and matrix protein (MA). Then, the virion envelope encloses the core and contains the envelope glycoprotein. In the case of retroviruses and lentiviruses, the envelope protein is made of the transmembrane (TM) and globular subunit (SU).

The Retroviridae family consists of spherical (80–120 nm) viruses containing a diploid, positive-sense ssRNA [7, 8] (Figure 1). The RNA genome is complexed with the nucleocapsid protein (NC), and bound to the reverse transcriptase (RT), integrase (IN) and protease (PR). The nucleocapsid is enclosed within a protein shell formed by capsid protein (CA). Then, matrix proteins (MA) surround this internal core, and interact with the virion lipid envelope, which incorporates viral envelope glycoprotein (ENV). ENV is formed by a TM (transmembrane) and SU (surface) domain, which binds to the cellular receptor and mediates virion entry.

## 3. The Retroviral Genome and Vector Engineering

Viruses from the Retroviridae family are usually divided in two groups, simple (such as Moloney mouse leukemia virus, MLV), and complex (such as lentiviruses) retroviruses. In any case, the genome organisation of both groups is similar in many aspects. In the two groups, the genome is organized from the 5′ to the 3′ end in *GAG*, *POL*, and *ENV* genes. While *GAG* encodes the structural proteins, *POL* encodes the reverse transcriptase, integrase, and protease, and *ENV* encodes the virus envelope glycoprotein responsible for virion entry into the target cell. All these enzymes are required for genome retro-transcription to cDNA, integration, and virion maturation [9]. The complex retroviruses additionally contain other accessory genes, which regulate viral replication, assembly, and pathogenesis [9–13]. Other additional genes of the complex retroviruses are important cis-acting sequences such as the RNA packaging signal (*ψ*) [14] required for genome encapsidation in virions [15], the polypurine tract (PPT) required for reverse transcription [16, 17], and the long-terminal repeats (LTRs) which contain the HIV promoter [18–20].

## 4. The Retroviral Life Cycle and Retroviral Vectors

**Figure 2:**
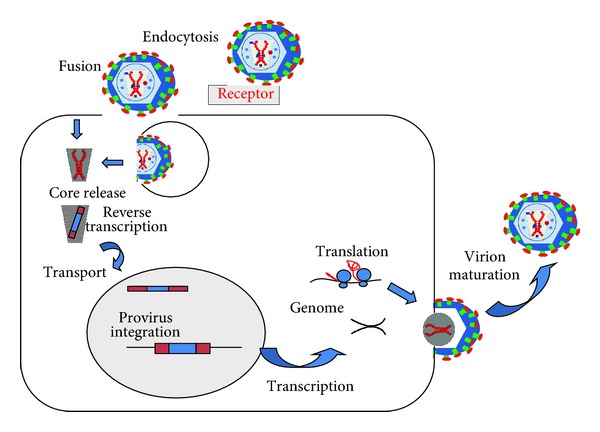
General life cycle of retrovirus/lentivirus. The lentivirus life cycle is schematically depicted in this Figure. First, the virion can enter the cell either by endocytosis of direct fusion with the cell membrane after binding to its specific receptor (top of the figure). Then, the retrovirus core is released (core release) and reverse transcription takes place. The core containing the cDNA virus genome is transported to the nucleus where it integrates into the host cell chromosome (provirus integration). From the provirus, transcription takes place leading to the transport of either full-length RNA genomes, or to mRNAs encoding the structural and enzymatic proteins (translation). The structural proteins package the full-length RNA genome during virion budding (see right of the figure), releasing infectious viruses following virion maturation.

The general retrovirus (including lentiviruses) life cycle is schematically depicted in Figure 2. The retrovirus virion binds to its specific cellular receptor through the surface unit of ENV. This interaction will determine the cell and tissue tropism of the particular virus. As retrovirus biology is fairly well-known, the identity of a wide number of these receptors is known. Examples of retrovirus cellular receptors are the murine cationic amino acid transporter and sodium/phosphate symporters for different strains of mouse leukemia virus (MLV) [21]. In the case of HIV-1, its receptor is the T cell lymphocyte marker CD4, and CXCR4/CCR5 as coreceptors [22–24].

Binding of SU induces a conformational change in ENV which exposes its fusion peptide, leading to fusion between the virion and cell membrane leading to the release of the retrovirus core to the cytoplasm. This release triggers the reverse-transcription reaction of the retrovirus genome, possibly by an increase in dNTP concentration [19, 25].

The viral core containing a single cDNA copy from the double stranded genomic RNA is transported to the nucleus and it is integrated into the host cell chromosomes following the activity of integrase (IN). While simple *γ*-retroviruses require the disappearance of the nuclear membrane during cell division, complex retroviruses (lentiviruses) can actively transport the core to the cell nucleus without requiring mitosis [26, 27]. Thus, retrovirus vectors only transduce cells during mitosis, while lentiviral vectors can transduce cells independently on their division status. This characteristic makes lentiviral vectors ideal for gene therapy of highly differentiated, postmitotic cells.

Once the cDNA is integrated into the host cell chromosome, it remains there as a provirus. This provirus will transcribe its genes from its LTR using the cellular RNA polymerase II and cellular transcription factors. This provirus will also remain integrated in the cell throughout its life and it will be transmitted to daughter cells following mitosis.

Following the standard transcription machinery, the mRNAs encoding the full-length viral RNA, and also the spliced versions encoding the viral proteins, are produced, transported out of the nucleus, and translated in the cytoplasm. Virus particles will then be assembled by specific interactions between the RNA genome and the GAG/GAG-POL polyproteins. This RNA packaging is coupled with viral assembly and released out of the cell by virion budding. This budding takes place at the cellular membrane, where the ENV glycoprotein is also incorporated to the budding virion [28–32]. Once the virion is released, the viral protease will exert its activities on the *GAG/GAG-POL* polyproteins, releasing the structural proteins and conferring infectivity to the newly formed virion [6, 33].

**Figure 3:**
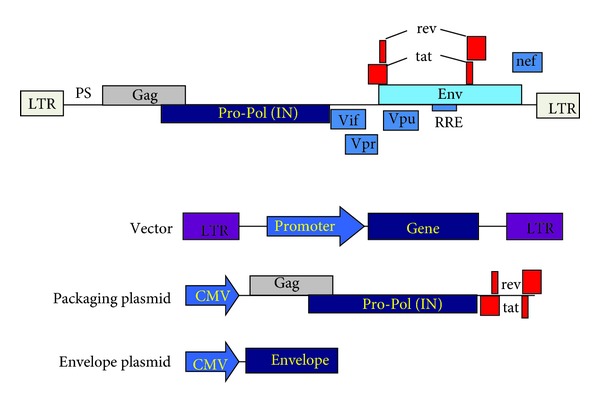
Engineering of the lentivector gene transfer system. The HIV-1 genome organisation is shown on the top, from 5′ to 3′, long-terminal repeats (LTR), packaging signal (PS), *GAG-PRO-POL*-In genes, envelope gene (*ENV*), and the accessory genes *Vif*, *Vpr*, *Rev*, *Tat*, *Nef*, and the Rev-response element (RRE). This genome is separated into three different plasmid constructs. The transfer vector (vector), that contains at least, the LTR, PS, and the internal promoter controlling the transcription of the gene of interest. The packaging plasmid, which leads to the expression of the *GAG-POL-PRO-IN*, *rev*, and *tat* genes under the control of the cytomegalovirus (CMV) promoter. Lastly, the envelope plasmid, which expresses the required envelope glycoprotein which will confer the tropism to the vector particle. Cotransfection of these three plasmids will lead to the production of lentivirus-like particles with the capacity of transducing target cells independently of their cell cycle.

For historical reasons, Moloney MLV has been extensively used for the development of integrative gene vectors [1]. To engineer a vector based on the MLV genome, the *GAG*, *POL*, and *ENV* genes are deleted to include either a gene of interest (under the transcriptional control of the virus LTR), or an expression cassette made of a promoter of choice with the gene of interest [1]. To generate the vector particles containing the transfer vector itself, the viral proteins GAG, POL and ENV are provided *in trans* in a packaging cell [1, 34]. In addition, several sequences are required *in cis* such as the 5′ and 3′ LTR, the packaging signal, and also others involved in in reverse transcription and genome integration. Cotransfection of these packaging plasmids will generate retrovirus/lentivirus-like particles with the packaged vector genome. Therefore, after cellular entry and integration, these vectors are unable to reassemble and produce infectious virions, as the lack the *GAG-POL* genes. Thus, once integrated, they will express the gene of interest that will be propagated to progeny cells. A scheme depicting the lentivector engineering system is shown in Figure 3.

While the advantages of simple retrovirus vectors are the lack of genome-encoded viral proteins and persistent gene expression after vector integration, they also present important limitations. The main ones are virion instability [35], relatively low titers [36], the inability to transduce quiescent cells [27, 37], and finally, insertional mutagenesis [38–40]. Interestingly, these shortcomings can be largely overcome by using lentivectors, mainly developed from human immunodeficiency virus (HIV)-1 [41–43]. However, the main advantage for the use of lentivectors is their capacity to transduce quiescent cells [27]. This crucial characteristic is mediated by nuclear localisation sequences present in the integrase protein, the matrix protein, vpr, and the PPT sequence [12, 17, 42]. The specific steps to generate lentivectors, their different “generations” and their biosafety are extensively described elsewhere [6, 44].

## 5. Immunological Tolerance

Our organisms are constantly in direct contact with an extensive variety of substances, particles, and living organisms of diverse origins. While many of these comprise a potential list of pathogens, the vast majority of them, including commensal bacteria, pollen, yeast, mites, and many types of chemicals are largely innocuous. Therefore, in the first instance the default immunological response to these antigens is tolerance and unresponsiveness. In addition to this, if an immune response is triggered by a potential pathogen/threat, autoprotective mechanisms exist to minimise collateral damage and loss of tolerance towards the organisms' own components (autoantigens).

**Figure 4:**
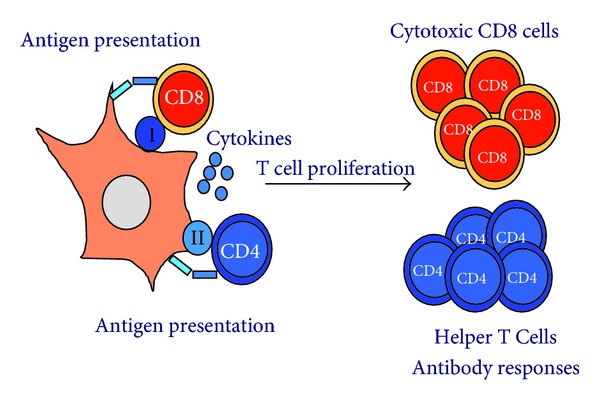
Antigen presentation and T cell responses. Activation of T cell responses by antigen presenting dendritic cells is shown in the figure. On the left, a DC is presenting antigenic peptides associated to MHC I (sphere containing “I”) or MHC II (sphere containing “II”) molecules. CD8 or CD4 T cells recognise these peptide-MHC molecules together with additional receptor-ligand interactions (costimulation, represented by bars connecting the DC with each T cell). These interactions accompanied by the presence of a wide range of cytokines will drive T cell proliferation (right). CD8 T cells will then differentiate into cytotoxic T lymphocytes, and CD4 T cells into helper T cells which will collaborate in raising antibody responses. T helper differentiation can lead to either immunological tolerance or different types of immune responses, such as a “Th1-” type (mainly cellular immunity) or a “Th2-” type (mainly humoral immunity).

Consequently, there are many key physiological mechanisms that maintain immunological tolerance. One of the most important is clonal deletion of autoreactive T lymphocytes in the thymus [45]. T lymphocytes expose T cell receptors (TCRs) on their surface that are specific for a particular antigen. When the cognate antigen is presented to these T cells, they strongly proliferate and activate their effector activities, for example, cytotoxicity (Figure 4). However, clonal deletion cannot eliminate all autoreactive T cells, or at least T cells specific to antigens that have never been present in the thymus. This fact is manifested in autoimmune disorders such as rheumatoid arthritis, multiple sclerosis, or diabetes, in which tolerance towards self-antigens is lost. These antigens are then recognised by B and T lymphocytes, which exert their effector activities with dramatic consequences [46–51].

Many of the autoreactive T lymphocytes with high affinity TCRs avoid clonal deletion and differentiate into natural Foxp3+ CD4 regulatory T cells, which are strongly immunosuppressive [45, 52]. In addition to natural Tregs, another immunosuppressive T cell differentiates in the periphery from naïve T lymphocytes. These inducible Tregs arise after antigen presentation in a “tolerogenic” context, mainly provided by tolerogenic dendritic cells (DCs) [53–55]. These tolerogenic DCs can be effectively targeted by gene therapy techniques using retrovirus and lentivirus vectors.

## 6. Tolerogenic DCs

DCs can either trigger effective immune responses or suppress them [56, 57]. Their acquisition of tolerogenic activities takes place under specific circumstances. In general terms, antigen presentation by immature DCs results in either Treg differentiation or T cell inactivation/apoptosis [58–61]. Tolerogenic DCs usually express low levels of surface major histocompatibility molecules (MHC) I and II and other costimulatory molecules of antigen presentation such as CD80, CD86, CD83, and ICAM I [53, 62–64]. The low surface expression of these molecules ensures that the interaction between the T cells and the antigen presenting cell does not lead to T cell activation [65].

There are multiple mechanisms by which DCs become tolerogenic and exert their immunosuppressive mechanisms. Some resident DCs, such as those in the gut and other mucosal tissues are strongly tolerogenic due to the presence of a wide range of immunosuppressive molecules. DCs can also become potently immunosuppressive after recognition of some microbial-derived antigens through their Toll-like receptors [66–68], lectin ligands, and immunosuppressive cytokines [53, 64, 66, 67, 69, 70].

Generally, all tolerogenic DCs secrete high amounts of immunosuppressive cytokines during antigen presentation, such as TGF-*β* or IL-10 [53, 63, 66, 67, 69–73]. In addition to this, tolerogenic DCs upregulate the surface expression of inhibitory costimulatory molecules, which inhibit T cell activation. This is the case of the T cell inhibitory receptor PD-1 ligand, PD-L1 [74–78]. PD-L2 is a second PD-1 ligand, which is specifically expressed on DCs and macrophages but its immunosuppressive capacities, are still under debate [79]. Other B7 family members are also immunosuppressive such as B7-H3 [80], B7-H4 [81], and VISTA [82].

Tolerogenic DCs can exert their immunosuppressive activities in a variety of mechanisms, and some of them include the upregulation of amino acid-metabolising enzymes such as arginase and indoleamine 2,3-dioxygenase (IDO) [83–88]. It is thought that consumption of essential amino acids by tolerogenic DCs depletes them from T cells, and their clonal expansion is arrested, becoming “inactivated”.

## 7. Genetic Modification of DCs by Retroviral and Lentiviral Vectors to Induce Immunosuppression and Tolerance

The capacity of DCs to “capture” viruses and being transduced with retrovirus and lentiviruses can be taken as an advantage to genetically modify them [62, 89, 90]. However, there are two main problems to overcome for efficient human DC modification. Firstly, monocyte-derived DCs are particularly difficult to transduce, as they possess an intracellular restriction factor to lentivirus infection [91]. Secondly, the process of DC transduction with these viral vectors can induce their phenotypical and functional maturation, particularly when high multiplicities of transduction are used [44, 90]. This is a strong obstacle to overcome if tolerance is to be achieved. These two problems can be circumvented by the incorporation of the simian immunodeficiency virus (SIV) Vpx into the lentivector capsid during production. Vpx can counteract the human DC restriction to lentivector transduction, resulting in the use of lower number of particles to achieve efficient DC modification [92]. However, if correctly harnessed, the immunosuppressive capacities of tolerogenic DCs can be exploited to induce therapeutic immunological tolerance. Particularly, lentivectors are especially suitable to modify DCs and render them tolerogenic. Lentivectors can introduce genes with immunosuppressive properties while simultaneously coexpressing an antigen of interest. Lentivector transduction leads to stable genome integration and long-lasting transgene expression. This last property is particularly important to induce tolerogenic DC differentiation and prolonged antigen presentation to T cells. Finally, as full-length transgenes can be expressed, it is not required to previously identify specific epitopes for particular MHC alleles. Thus, lentivector vaccines could be readily applied to patients without the need of MHC typing.

**Figure 5:**
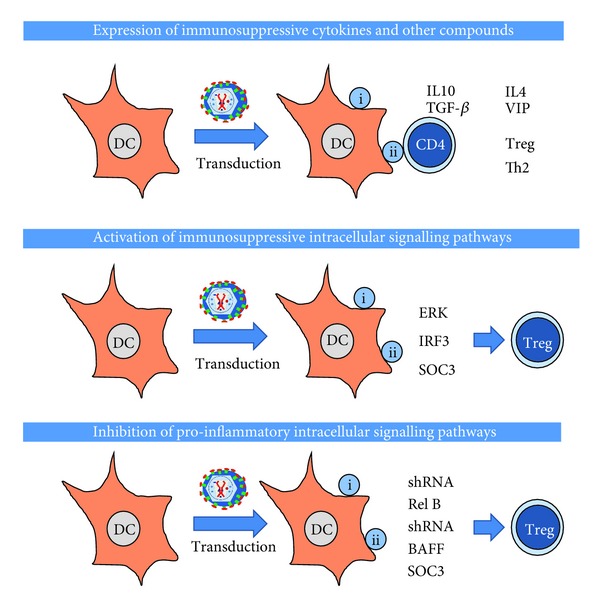
Main strategies for the induction of immunological tolerance by genetic modification of DCs using retroviral/lentiviral vectors. In this scheme, the most utilised strategies for the induction of immunological tolerance using gene modification of DCs are shown. On top, using lentiviral or retroviral vectors, the expression of immunosuppressive cytokines in DCs can induce the differentiation of antigen-specific Tregs, or in some cases, Th2 cells. In the middle, lentivectors can be used to express constitutive activators or immunosuppressive intracellular signalling pathways leading to the differentiation of suppressive T cells. On the bottom, lentiviral vectors can either deliver short hairpin RNAs targeted towards proinflammatory pathways such as NF-*κ*B, proinflammatory receptors such as BAFF, or inhibitors of cytokine signalling such as SOC3. All these strategies also lead to the development of suppressive T cells. Not shown in any of these schemes, all strategies lead to differentiation of tolerogenic DCs which will either prevent the expansion of cytotoxic CD8 T cells, or induce their apoptosis/anergy.

The most straightforward procedure to differentiate tolerogenic DCs is the delivery of potent immunosuppressive cytokines (Figure 5). Retroviral and adenoviral vectors have been successfully used for the treatment of inflammatory diseases. This is the case of constitutive expression of TGF-*β* using adenovirus vectors, leading to inhibition of immune responses and prolonged DC survival [93]. Likewise, constitutive expression of IL-4 in modified DCs inhibited collagen-induced arthritis in mouse models [94]. Retroviral delivery of viral IL-10 to DCs, naturally encoded by the Epstein-Bar virus, strongly inhibited their ability to stimulate T cells *in vitro* [73]. Similarly to retroviral transduction, lentivectors have also been used to express IL-10. These DCs could efficiently inhibit an OVA-dependent model of experimental asthma in mice. These modified DCs expanded IL-10-expressing Foxp3+ Tregs. Interestingly, IL-10 expression by the host Tregs was required to establish tolerance, rather than IL-10 expression by DCs [95].

Apart from the expression of immunosuppressive cytokines, tolerogenic DCs can be differentiated by targeting specific intracellular signalling pathways (Figure 5). It is well known that mitogen activated protein kinase (MAPK) extracellularly regulated kinase (ERK) activation in DCs is strongly immunosuppressive [63, 66, 96–100]. In fact, sustained MAPK ERK activation can be achieved by expression of constitutively active forms of its upstream MAPK MEK1/2 [97, 99]. Lentivector delivery of a constitutively active MEK1 mutant to DCs resulted in immature DCs with CD40 downmodulation and expression of bioactive TGF-*β* [53, 63]. These ERK-activated DCs differentiated antigen-specific Foxp3 Tregs *in vitro* and *in vivo* [53]. These Tregs strongly expanded *in vivo* after a second antigen encounter in inflammatory conditions, reinforcing antigen-specific tolerance, which controlled inflammatory arthritis in a mouse model [53]. Other intracellular signalling pathways have also been exploited to induce antigen-specific tolerance. This is also the case of constitutive activation of the type I IFN signalling pathway in DCs. Lentivector expression of a constitutively active IRF3 mutant, induced expression of IL-10 by modified DCs possibly through interactions with the TLR adaptor molecule MYD88 [63]. These modified DCs also expanded antigen-specific Foxp3 Tregs *in vivo*, which inhibited effector T cells. This is not surprising as the type I interferon pathway is immunosuppressive in certain contexts [101], and both IFN-*β* and IL-10 secretion share a common signal transduction pathway [63, 101, 102]. Thus, IFN-*β* is administered in patients of multiple sclerosis [103, 104]. Very interestingly, direct lentivector vaccination achieves *in vivo* transduction of the sufficient number of DCs to induce tolerance, which was effective for at least one month [53, 63, 90, 105, 106]. A potential drawback of all these strategies is the limited lifespan of transduced DCs. However, this can be overcome by intravenous administration of lentivectors, which can transduce DC precursors present in tissues leading to long-term transgene expression [107, 108].

Another strategy based on modification of intracellular signalling is the inhibition of proinflammatory signalling pathways (Figure 5). In this way, their inhibition can in some circumstances induce tolerance. A classic example is the inhibition of the NF-*κ*B pathway, a well-known proinflammatory route [62]. Consequently, Rel-B silencing by specific shRNAs prevented DC maturation after TLR stimulation, a strategy which was successfully used for the treatment of autoimmune myasthenia gravis in mice [109]. Conversely, the targeted activation of endogenous negative feedback mechanisms of proinflammatory pathways can also be exploited. When the suppressor of cytokine signalling 3 (SOCS-3) was overexpressed in DCs, these modified DCs exhibited impaired proinflammatory signalling [110]. These modified DCs showed a markedly reduced expression of classical proinflammatory cytokines such as IFN-*γ*, IL-12, and IL-23. They also showed an enhanced IL-10 secretion. Overall, these SOC-3 expressing DCs could effectively inhibit experimental autoimmune encephalomyelitis (EAE) in mice, a model of human multiple sclerosis [110].

In some cases, the pathogenic antigen leading to autoimmune disease is unknown in humans. This is clearly the case in rheumatoid arthritis. Even then, lentivectors can also be used to suppress autoimmune disorders without the direct targeting of the pathogenic antigen. Therefore, administration of a B cell activating factor (BAFF)-specific siRNA in the inflamed joint was sufficient to treat experimental collagen-induced arthritis [111–113]. These lentivectors preferentially transduce DCs in the inflamed joint without the need of modifying their tropism *in vivo*. Expression of this BAFF siRNA interfered with DC maturation and inhibited Th17 differentiation [112]. Retroviral modification of T cells can also be performed to achieve tolerance in diseases with unknown (uncharacterised) pathogenic antigens. For example, the introduction of an OVA-specific TCR in Foxp3+ Tregs by retroviral transduction modified their specificity towards OVA. The administration of OVA in the inflamed joints in a model of inflammatory arthritis in mice in which these OVA-Treg cells were adoptively transferred allowed the suppression of inflammation and bone destruction. This circumvented the need of targeting the pathogenic arthritogenic antigen [114]. In a similar fashion, OVA-specific Tregs generated by ERK-activated (by lentivector transduction) OVA-expressing DCs achieved the same end [53, 63].

Lentivectors can also deliver a wide range of immunosuppressive mediators, such as the vasointestinal peptide (VIP). VIP expression of lentivector-transduced DCs effectively inhibited DC maturation and resulted in expression and inhibition of immunosuppressive and proinflammatory cytokines, respectively. Their therapeutic efficacy was mediated by differentiation and expansion of Foxp3+ Tregs [115]. Basically, the same strategy was successfully used for the treatment of EAE and in the coecal ligation and puncture (CLP), models for multiple sclerosis and sepsis in humans, respectively [106]. Even viral proteins with immunosuppressive properties can be expressed by lentivectors to induce tolerance [116].

## 8. Induction of Immunological Tolerance by MicroRNA-Tagging

A major problem in lentivector gene therapy is that its direct administration *in vivo* raises a strong transgene-specific immune response. This is desirable to boost immunity for the treatment of infectious diseases and cancer [63, 90, 117–120]. However, this property of lentiviral vectors is very detrimental for gene therapy of genetic and metabolic disorders. Transgene-specific immune responses limit the survival of corrected cells, and therefore, their therapeutic activities [121–123]. A very elegant strategy to prevent trangene-specific immune responses was achieved by miRNA tagging [124] (Figure 5).

MicroRNAs (miRNAs) comprise a collection of non-coding RNAs, which form part of a posttranscriptional regulatory system of gene expression. MiRNAs contain small sequences of 20–24 nt, termed siRNAs, which are partially complementary to endogenous mRNAs. These siRNAs can inhibit gene expression by mainly (but not exclusively) inducing mRNA degradation. This endogenous regulatory system was also exploited to prevent an immune attack against genetically corrected cells using lentiviral vectors as gene carries. It was shown that transgene expression in professional antigen presenting cells was responsible for antigen-specific T cell responses. These T cells exert their cytotoxic activities towards transgene-expressing cells. Therefore, to avoid T cell responses, the expression of the transgene was prevented specifically in professional antigen presenting cells by introducing a sequence target for the haematopoietic-specific miRNA 142 3p in the transgene [125]. Thus, the mRNA coding for the transgene would be degraded in cells from the hematopoietic lineage (lymphocytes, granulocytes, macrophages, and DCs), but not in cells from other lineages. 142 3p-tagged lentivectors could then be intravenously administered without raising transgene-specific immune responses leading to long-term transgene expression in hepatocytes [125]. Rather than immunological silencing, this strategy induced transgene-specific tolerance by expansion of Foxp3+ Tregs [126]. The authors of the study showed that transgene expression in hepatocytes was required to expand Tregs [126]. miRNA 142 3p-tagging was effectively used to express factor IX in liver, leading to correction of experimental haemophilia B [127].

## 9. Biosafety Considerations on the Application of Retroviral and Lentiviral Vectors

Biosafety considerations are clearly a priority when applying new experimental therapies to human disorders that may be tackled using more conventional approaches. This is the case of some autoimmune/inflammatory disorders such as rheumatoid arthritis or diabetes. Biosafety concerns of using retrovirus and lentivirus vectors have been particularly highlighted after the first clinical application of MLV-based retrovirus vectors for the treatment of X-linked SCID and chronic granulomatous disease. In the case of the X-SCID trials, a significant number of treated children developed leukemia, possibly linked to insertional mutagenesis by transcriptional upregulation of proto-oncogenes [38, 128–130]. The removal of the proviral LTRs can partially solve this problem (self-inactivating lentivectors [20, 131, 132], since the viral enhancers that they contain strongly up-regulate proto-oncogenes and also lead to aberrant transcription and alternative splicing [128, 129, 133, 134]. All these genotoxic effects could also be prevented, at least for the induction of tolerance, using nonintegrating lentivectors (NILVs). These lentivectors can be generated straightforwardly by introducing inactivating mutations in the integrase coding region, or integrase-binding sites in the transfer vector [118, 135]. Consequently, the vector remains in the nucleus as an episome, and long-term expression is achieved in non-dividing cells, suitable for DC genetic modification. NILVs have been shown to be effective for gene modification of retina, muscle, and brain [135–138], and they have been already used for vaccination [108, 118, 139, 140].

Genotoxic effects are possibly more severe in poorly differentiated cells, rather than in terminally differentiated, postmitotic cells. Therefore, another way to increase biosafety is to physically target the lentivector particle to the desired cell/tissue. This can be achieved by pseudotyping of the retrovirus/lentivirus vector particle. The envelope glycoprotein (ENV) present on the particle's surface determines the specific recognition of the target cell. Then, after ENV binding to its appropriate cellular receptor, the vector particle gains entry into the cell. Retrovirus and lentivirus vectors can acquire a large number of different envelope glycoproteins as they bud at the cellular membrane from the producer cell. Interestingly, these viral particles exhibit the natural tropism conferred by the incorporated glycoprotein [141, 142]. The most used envelope glycoprotein for lentivector pseudotyping is the vesicular stomatitis virus glycoprotein (VSV-G) [41, 42, 143, 144]. Pseudotyping with VSV-G presents many advantages, including particle stability and high titer vector preparations [29]. Importantly, VSV-G confers virus vector particles with a very broad host cell range, which includes mouse and human DCs [10, 29, 145]. Therefore, potentially, the restriction of lentivector tropism may result in safer *in vivo* gene delivery and enhancement of the therapeutic effects by a reduction in the lentivector dose.

Retroviral and lentiviral vectors have been successfully pseudotyped with a wide range of different heterologous viral proteins [146, 147]. As examples, mouse leukemia virus amphotropic (MLV-A), gibbon ape leukemia virus (GALV), and feline endogenous retrovirus (RD114) envelopes [31, 148–153]. Lentivector pseudotyping with alphavirus envelopes confers specific tropism towards mouse and human DCs [154, 155]; Interestingly, baculovirus gp64 confers transduction capacity to hepatocytes but not cells from the immune system, a property that can be exploited for the induction of immunological tolerance [156, 157]. This property can be exploited to prevent transgene-specific immune responses.

Pseudotyping with measles virus H/F ENV envelope glycoproteins confers transduction capacity to resting human B and T cells, with the possibility of targeting these immune cell types for the induction of tolerance [30, 158].

Additionally, lentivectors can also be pseudotyped with modified viral glycoproteins, conferring new tropisms for the vector particles. Just as an example, specific gene targeting *in vivo* to DCs was successfully accomplish by introducing selected mutations in the Sindbis virus envelope proteins E1/E2, to enhance binding to DC-SIGN [159, 160].

## 10. Conclusions

Since retroviral vectors have proven to be efficient systems to deliver genes of interest into target cells they are being used for gene therapy and the development of gene vaccines. What makes them good systems is that they are devoid of viral proteins, and stably incorporate into target cells. Lentiviral vectors, developed from complex retroviruses, have properties that overcome several limitations of simple retroviral vectors, including higher virion stability and titers and a lower frequency of insertional mutagenesis. Especially important for immunotherapy is the fact that they can transduce highly differentiated cells such as DCs [6].

DC transduction with retroviral vectors causes their phenotypical and functional maturation. While this is an advantage when treating infectious disease or cancer it has the adverse effect when immunological tolerance is the desired outcome, as it is in the case of treatment of autoimmunity.

Lentivectors delivering immunosuppressive mediators to inhibit the maturation of transduced DCs could be designed to induce tolerance. On the other hand, there are different types of matured DCs and not all of them induce an immune response. There are tolerogenic DCs in the periphery that provide tolerogenic signals to autoreactive T cells with high-affinity TCRs that cause them to differentiate into inducible Treg's [53–55]. These tolerogenic DCs can be used to achieve immunological tolerance. To differentiate tolerogenic DCs there are three general (most used) methods; the delivery of potent immunosuppressive cytokines, targeting of intracellular signaling pathways leading to a tolerogenic phenotype (MAPK/ERK activation), and the inhibition of proinflammatory signaling pathways (NF-*κ*B pathway). What makes lentivector transduction of DCs an especially elegant method is the fact that it can be applied without previous MHC typing as the DC will automatically present antigens from the transgene. The combination of delivering immunosuppressive properties while coexpressing an antigen of interest will prime a tolerogenic response towards this antigen.

Gene therapy by lentivectors, while promising, had limited success due to the fact that these vectors induce a strong transgene-specific immune response *in vivo* that limits the survival of corrected cells and their therapeutic activities. To overcome this hurdle, miRNA tagging could be exploited to prevent transgene expression in professional antigen presenting cells by introducing a sequence target for specific miRNAs present in these cells, or in certain maturation stages.

The use of retroviral vectors would in principle seem more suitable to diseases where the immune system needs to be boosted, such as cancer. But due to the elegance of our immune system that can induce immunity or tolerance, its modulation using gene therapy approaches may prove to be an effective system to counter the overprotective reactions of our immune system, which may give rise to autoimmune disease.
